# Emerging understanding of apoptosis in mediating mesenchymal stem cell therapy

**DOI:** 10.1038/s41419-021-03883-6

**Published:** 2021-06-09

**Authors:** Yu Fu, Bingdong Sui, Lei Xiang, Xutong Yan, Di Wu, Songtao Shi, Xuefeng Hu

**Affiliations:** 1 Fujian Key Laboratory of Developmental and Neural Biology & Southern Center for Biomedical Research, College of Life Sciences, Fujian Normal University, Fuzhou, Fujian 350117 China; 2 South China Center of Craniofacial Stem Cell Research, Guanghua School and Hospital of Stomatology, Sun Yat-sen University, Guangzhou, Guangdong 510055 China; 3 Research and Development Center for Tissue Engineering, The Fourth Military Medical University, Xi’an, Shaanxi 710032 China

**Keywords:** Apoptosis, Stem-cell research, Translational research

## Abstract

Mesenchymal stem cell transplantation (MSCT) has been recognized as a potent and promising approach to achieve immunomodulation and tissue regeneration, but the mechanisms of how MSCs exert therapeutic effects remain to be elucidated. Increasing evidence suggests that transplanted MSCs only briefly remain viable in recipients, after which they undergo apoptosis in the host circulation or in engrafted tissues. Intriguingly, apoptosis of infused MSCs has been revealed to be indispensable for their therapeutic efficacy, while recipient cells can also develop apoptosis as a beneficial response in restoring systemic and local tissue homeostasis. It is notable that apoptotic cells produce apoptotic extracellular vesicles (apoEVs), traditionally known as apoptotic bodies (apoBDs), which possess characterized miRnomes and proteomes that contribute to their specialized function and to intercellular communication. Importantly, it has been demonstrated that the impact of apoEVs is long-lasting in health and disease contexts, and they critically mediate the efficacy of MSCT. In this review, we summarize the emerging understanding of apoptosis in mediating MSCT, highlighting the potential of apoEVs as cell-free therapeutics.

## Facts

- In the human body, 50–70 billion cells die every day, during which plenty of apoptotic extracellular vesicles (apoEVs) are produced and are involved in tissue homeostasis maintenance and disease development.
- Infused mesenchymal stem cells (MSCs) disappear soon in the recipients, which are further revealed to undergo extensive apoptosis.
- Transplanted apoptotic MSCs and apoEVs interact with recipient cells, which improve tissue regeneration and immunomodulation.

## Open questions

- Do endogenous MSCs autonomously execute apoptosis and result in abundant apoEV production that contribute to systemic and local tissue homeostasis maintenance?
- Whether infused MSCs undergo other cell death processes attributed to diverse physiological and pathological contexts, such as the autophagic cell death, the necroptosis and the pyroptosis?
- Whether EVs produced by multiple cell death processes have differential regulatory and therapeutic effects?

## Introduction

Since mesenchymal stem cells (MSCs) were originally discovered in the early 1970s, these primitive cells have been known to give rise to multilineage descendants while retaining the capacity to self-renew^1–3^. In recent years, increasing understanding of these cells as crucial contributors to organogenesis and immunomodulation has led to the development and application of preclinical and clinical studies based on MSC transplantation (MSCT)^4–9^. While the therapeutic effects of MSCT on degenerated organs/tissues and immune disorders have been studied in some detail, the mechanisms by which transplanted MSCs interplay with recipients to provoke therapeutic cascades after administration remain less defined, resulting in bottleneck problems on the path toward controllable and precise therapies. Along with various therapeutic cytokines, MSCs release multiple extracellular vesicles (EVs), which are membrane-bound structures of endosomal origin or shed from the plasma membrane^10,11^. As carriers of bioactive molecules and organelles transferred to recipient cells, EVs possess specific biological functions and have immense effects in MSCT^10,12^. Accumulating studies have reported that MSC-derived EVs exert beneficial effects in various disease models through transferring proteins and microRNAs (miRNAs), constituting one paracrine mechanism of MSCT^13–16^.

Apoptosis, a physiological and autonomous clearance process used by an organism to remove unwanted cells, was also first described in the early 1970s and subsequently found to play significant roles in development, tissue homeostasis, aging, and pathogenesis^17–20^. During the execution of apoptosis, apoptotic EVs (apoEVs), originally known as apoptotic bodies (apoBDs), are formed by membrane blebbing or protrusion with specific intracellular content distribution, and have emerged as regulators of multiple biological processes rather than mere debris^21^. In particular, apoEVs have been shown to critically regulate T-cell and macrophage immune function, as well as promote tissue recovery including skin regeneration and vascular protection^22–26^. Notably, increasing evidence has suggested that transplanted MSCs undergo extensive apoptosis, during which the released apoEVs serve as indispensable therapeutic mediators^27–30^. Functioning through engulfment or dynamically interacting with recipient cells, apoEVs exert regulatory effects based on a fine-tuned molecular network^24,30^. Importantly, direct delivery of apoptotic MSCs or apoEVs produced by allogeneic apoptotic MSCs has further been revealed to possess advantages over viable MSCs^31,32^. Accordingly, MSC-derived apoEV transplantation holds the promise of counteracting various diseases including myocardial infarction (MI), osteoporosis, graft-versus-host disease (GvHD), colitis, and more^22,30–34^. Here we review the cutting-edge knowledge regarding apoptosis and apoEVs in mediating MSC therapy.

## Historical perspectives on MSCs and MSCT

MSCs are non-hematopoietic stromal cells which were originally isolated and identified in postnatal mammalian bone marrow (BMMSCs) by Friedenstein et al.^2^. They possess plastic adherence and clonogenic properties with multilineage differentiation capabilities in vitro^3^. Enlightened by BMMSC discoveries, a series of MSCs were isolated and identified from a variety of mammalian tissues including the adipose tissue, umbilical cord, tendons, and the orofacial region^35–41^. These MSCs from other sources not only display features typical of BMMSCs but also exhibit the functional characteristics associated with their tissue-specific origins and locations^42–46^. In addition to their self-renewal and differentiation potential, MSCs are further characterized by potent immunomodulatory properties. For example, they suppress proliferation and activation of immune cells, particularly T cells^9,47–52^. Several classical markers have been generally used to identify human BMMSCs by their surface antigens, including but not limited to CD105, CD146, CD271, and STRO-1, while CD11b, CD31, CD34, and CD45 serve as negative markers^53–56^. Other tissue-specific MSCs, such as dental pulp stem cells (DPSCs), are derived from neural crest cells in early head development and express neurovascular-associated markers including neuron glia 2 (NG2) and alpha-smooth muscle actin (α-SMA), as they contribute to neurogenesis and angiogenesis^57,58^. Furthermore, functional markers for certain MSC subpopulations, such as nestin, Gli1, leptin receptor (LepR), and programmed cell death 1 (PD1), have been revealed to control MSC proliferation and differentiation in vivo^57,59–61^.

In light of their self-renewal, multilineage differentiation, and immunoregulatory properties, MSCs have been widely used as cellular therapeutics in tissue regeneration and treatment of immune disorders, which has prompted a spectrum of clinical studies. The first clinical therapeutic application of allogenic MSCs dates back to the early 2000s with Horwitz’s study in which six children with severe osteogenesis imperfecta received transplantation of allogeneic BMMSCs^4^. The results demonstrated therapeutic effects including acceleration of the tissue growth during the first 6 months post-infusion^4^. Subsequently, Le Blanc et al. have accomplished the first clinical trial showing significant efficacy of MSCs in treating human GvHD^5^. To date, many clinical trials aimed at tissue regeneration have been initiated or accomplished, applying MSCT to treat ischemic heart failure, osteonecrosis, osteoarthritis, and more^62–64^. Moreover, in situ transplantation of DPSCs has been applied for dental pulp regeneration in humans, the success of which may be attributed to their capacity to give rise to neurovascular tissue as noted above^65^. In parallel, extensive clinical trials for treatment of immune disorders have been conducted, such as in GvHD, systemic lupus erythematosus (SLE), and multiple sclerosis^66–68^. To date, more than 400 studies on MSC immunomodulation have been registered in clinical trial databases. Collectively, these trials establish that the recognition of MSC-specific characteristics represents an important basis for future clinical translational medicine.

## Therapeutic mechanisms of MSC transplantation

**Fig. 1:**
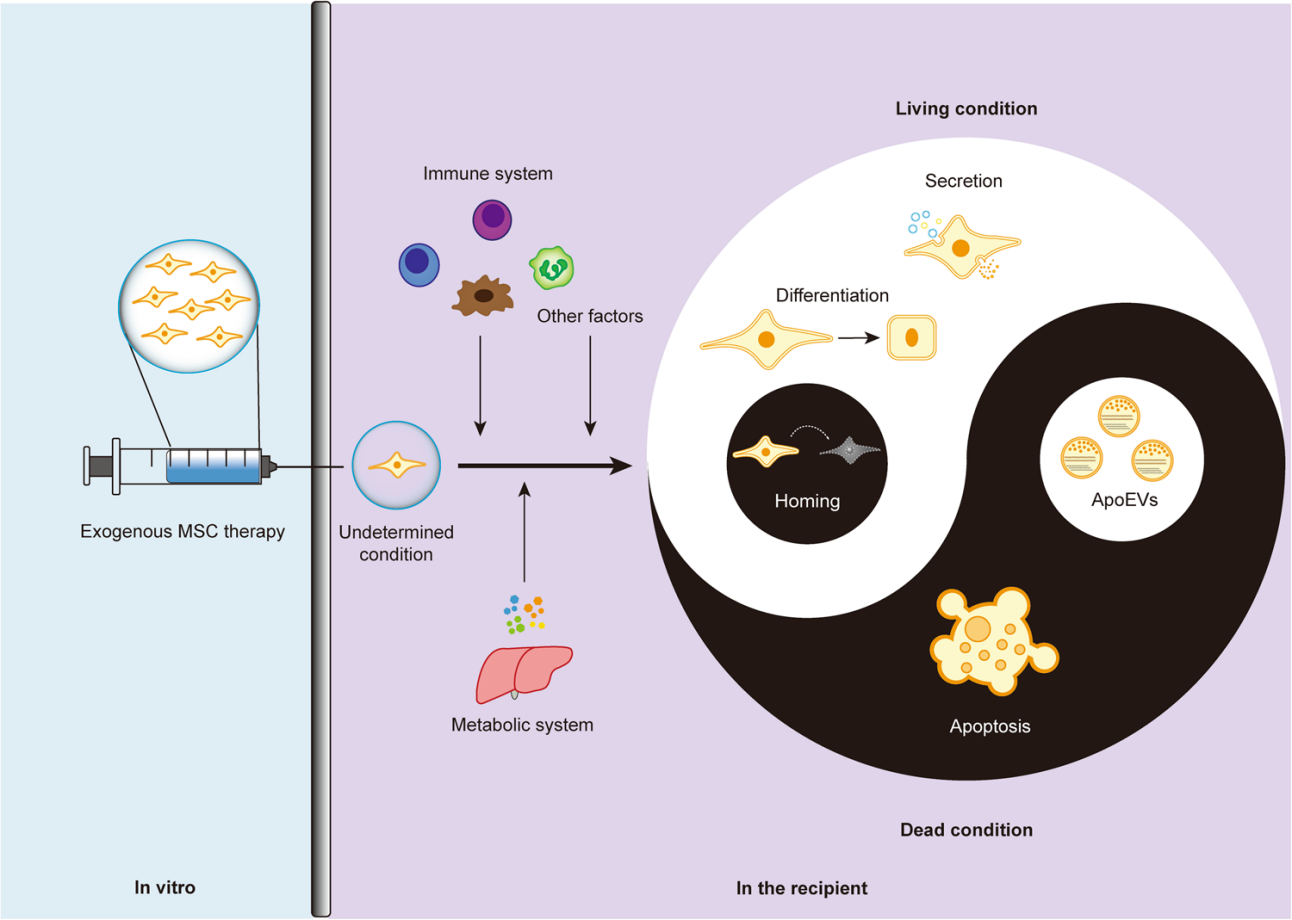
Live-dead decision of MSCs in therapy. After infusion, exgenous MSCs in the undetermined condition confront with the stimuli of multiple factors inculding immunological, metabolic, and other cues. Subsequently, the MSCs can be alive to exert therapeutic effects based on differentiation and secretion, which also can undergo apoptosis to regulate immune responses. ApoEVs have been demonstrated to be a noval and potent therapeutic in translational medicine. The above condition of live-dead decision for infused MSCs based on current recognitions has emerged as a representative paradigm of Taichi.

While MSCT has exhibited extensive biological effects that promote regenerative repair and immunoregulation, the cellular and molecular mechanisms underlying the potential therapeutic interplay between transplanted MSCs and recipient components remain elusive. Through direct effects based on engraftment and differentiation, as well as indirect effects based on paracrine mechanisms including cytokines and EVs, MSCs exert immense therapeutic efficacy^13–16^ (Fig. 1). It has been widely reported in MI that intravenous-infused MSCs engraft and differentiate into cardiomyocytes while recruiting endogenous cardiac stem cells, which attenuate the progressive deterioration of the heart and improve cardiac function^6,7,69^. In parallel, transplanted MSCs have dynamic interactions with the local stem cell niche, which also contributes to the tissue recovery^70^. While the above findings have demonstrated that MSCT repairs tissue injury in various diseases *via* regulating the engrafted tissue, answers to the questions of how MSCs engraft in recipient tissues and how long they remain in these recipient tissues remain elusive. It has been proposed that homing of transplanted MSCs relies on recruitment by endogenous cells^71^, but there has been no direct evidence to prove that the engrafted MSCs are still alive to exert effects (Fig. 1). Intriguingly, further studies have reported that transplanted MSCs are trapped in the lung and become undetectable within 24 h post-injection, which subsequently undergoing extensive apoptosis over the short term^6,28,30^ (Fig. 1). Accordingly, revealing the bona fide mechanisms of MSCT will be highly significant for improving strategy for tissue regeneration and homeostatic maintenance.

With regard to paracrine mechanisms, pioneering studies by Gnecchi et al. have shown that injection of conditioned medium (CM) of MSCs remarkably improves cardiac performance^72,73^. Subsequently, increasing evidence has suggested that infused MSCs secrete a serious of cytokines, including TNF-stimulated gene 6 protein (TSG-6), prostaglandin E2 (PGE2), insulin-like growth factor 2 (IGF-2), indoleamine 2,3-dioxygenase (IDO) metabolite kynurenine, vascular endothelial growth factor (VEGF), and basic fibroblast growth factor (bFGF), among others, to regulate recipient cells in immunomodulation, angiogenesis, and migration^6,28,74–79^. Furthermore, as MSCs secrete a large amount of EVs, including exosomes (with diameters in the range of 30–100 nm) and microvesicles (diameters within the range of 50–1000 nm), EV release has been increasingly recognized as a critical mechanism for the transfer of bioactive molecules in MSC therapy^10,21^. It has been reported that infused MSCs, through exosomes transferring Fas protein, modulate the intra-/extracellular balance of miR-29b in recipient stem cells and recover DNA methyltransferase 1 (Dnmt1)-mediated Notch promoter hypomethylation and Notch signaling activation, indicating epigenetic regulation of recipient stem cells by MSCT-mediated paracrine mechanisms^80^. It has further been documented that miR-151-5p secreted within exosomes by donor MSCs can be transferred to endogenous MSCs in systemic sclerosis mice to inhibit interleukin 4 receptor α (IL4Rα) expression and block mammalian target of rapamycin (mTOR) pathway activation, which rescues endogenous MSC functional defects in treating osteoporosis^81^. MSCT-mediated exosomal or microvesicle transfer of functional proteins and non-coding RNAs has been widely reported in treating many other diseases, including MI, acute lung injury, and experimental colitis^13,15,82–84^.

Considering all these studies, the bona fide regulatory mechanisms of MSCT have increasingly been revealed, which has further shaped our understanding of the behaviors of MSCs in translational medicine. While there is a lack of long-term engraftment after MSCT, there is also potent secretion of EVs. Whether and how these two processes are linked in transplanted MSCs remains an intriguing question. In this regard, recent studies on release EVs, particularly in the course of MSC apoptosis, have provided a new perspective on MSC therapy, as stated below.

## Apoptosis in organismal homeostasis and therapeutic processes of MSCT

As the most prominent mode of programmed cell death (PCD), apoptosis has been recognized as a physiological process that is widely involved in development, tissue homeostasis, aging, and pathogenesis^85–87^. During apoptosis, a cell undergoes a serious of well-characterized morphological changes including cytoplasmic shrinkage, membrane blebbing or protrusion, and nuclear condensation^17,20,85,87^. Subsequently, it has been revealed that active caspases cleave Rho effector protein ROCK1, which generates a truncated kinase with biological activity for actin-myosin remodeling and cell contractility^88^. Then, the cellular membrane gradually protrudes accompanied by blebbing and is fragmented in the final formation of apoptotic debris and apoEVs^21^.

Apoptosis is closely correlated with the immune balance of an organism. Because immune systems would be overactivated if immunogenic intracellular materials were released, it is necessary to clear apoptotic cells or apoEVs quickly enough to prevent secondary necrosis and thereby remain tissue homeostasis^19,89^. Furthermore, apoptosis has proven to be critical for attenuation of autoimmune reactions, not only by directing phagocytic cells into an anti-inflammatory phenotype, but also by regulating adaptive immune responses mediated by T cells and B cells^22,90–93^. Importantly, inefficient engulfment of endogenous apoptotic cells can cause a variety of autoimmune diseases, such as SLE, severe anemia, and chronic arthritis^94–98^.

The delicate equilibrium between stem cell-mediated proliferation (i.e., compensatory proliferation) and the neighboring stem or somatic cell apoptosis plays an indispensable role in tissue regeneration after injury^99^. Studies have reported that WNT and c-Jun amino-terminal kinase (JNK) signaling induced by surrounding apoptotic stimuli contribute to compensatory proliferation^100,101^. Recent evidence has also shown that apoptotic epithelial stem cells facilitate adjacent stem cell proliferation by caspase-dependent production of WNT8a-containing apoEVs, suggesting that the plasticity of stem cells enables them to adapt to tissue homeostatic and regenerative needs upon sensing apoptotic signaling^102^. Furthermore, deletion of the pro-apoptotic protein ARTS in intestinal stem cells enhances WNT signaling and stimulates augmented cell proliferation in the tissue, indicating dynamic regulation of tissue homeostasis by apoptotic signaling interactions within the stem cell niche^103^. Apoptotic signaling has also been identified as crucial to maintaining hepatic and neural tissue regeneration^104–106^. In addition to the contribution to cell proliferation, apoptotic cells can trigger non-autonomous apoptosis of surrounding cells *via* production of tumor necrosis factor (TNF) homolog Eiger to activate JNK pathway in *Drosophila*^107^, indicating the complex nature of the roles of apoptosis plays in tissue maintenance.

In MSCT, apoptosis is also an important biological process that has gradually been noticed (Fig. 1). It has been reported that infused human MSCs (hMSCs) are trapped and disappear in the lung, whereas the anti-inflammatory protein TSG-6 is upregulated in the lung to prevent injury^6^. It has further been documented that infused MSCs decrease markedly in tissues with extensive apoptosis within 24 h, which intriguingly promotes their secretion of TSG-6 to prevent hypertrophic scar formation^28^. As shown by the evidence in vitro, MSCs activated complement system and suffered injury after serum contact, while are further proposed that infused MSCs are involved in interaction with the recognition and attack of complement in vivo^108^. Apoptosis of infused MSCs can also be induced by pro-inflammatory T cells *via* interferon-gamma (IFN-γ) and TNF-α^27^. Moreover, perforin-dependent apoptotic execution of transplanted MSCs has been demonstrated to be essential for the initiation of MSC-induced immunosuppression, which has been further confirmed in patients with GvHD: only those with high cytotoxic activity against MSCs respond to MSC infusion^29^. Importantly, release of apoEVs by infused MSCs has recently been revealed as a novel mechanism of MSC communication with the recipient microenvironment to promote tissue immunoregulation and regeneration^23,30^ (Fig. 1). Other than apoptosis of the infused MSCs per se, it is notable that they also induce recipient T-cell apoptosis *via* the Fas ligand (FASL)-FAS pathway^109^. The apoptotic T cells are then phagocytosed by macrophages and induce Treg upregulation to establish an immune balance, which contribute to autoimmune suppression and amelioration of pathological symptoms in colitis and systemic sclerosis^109^. Given the importance of apoptosis to MSCT, further elucidation of the mechanisms by which apoptosis contributes to MSCT is an intriguing and important matter, which would open a new window for effective application of MSCT.

## Production and functionality of apoEVs

**Fig. 2:**
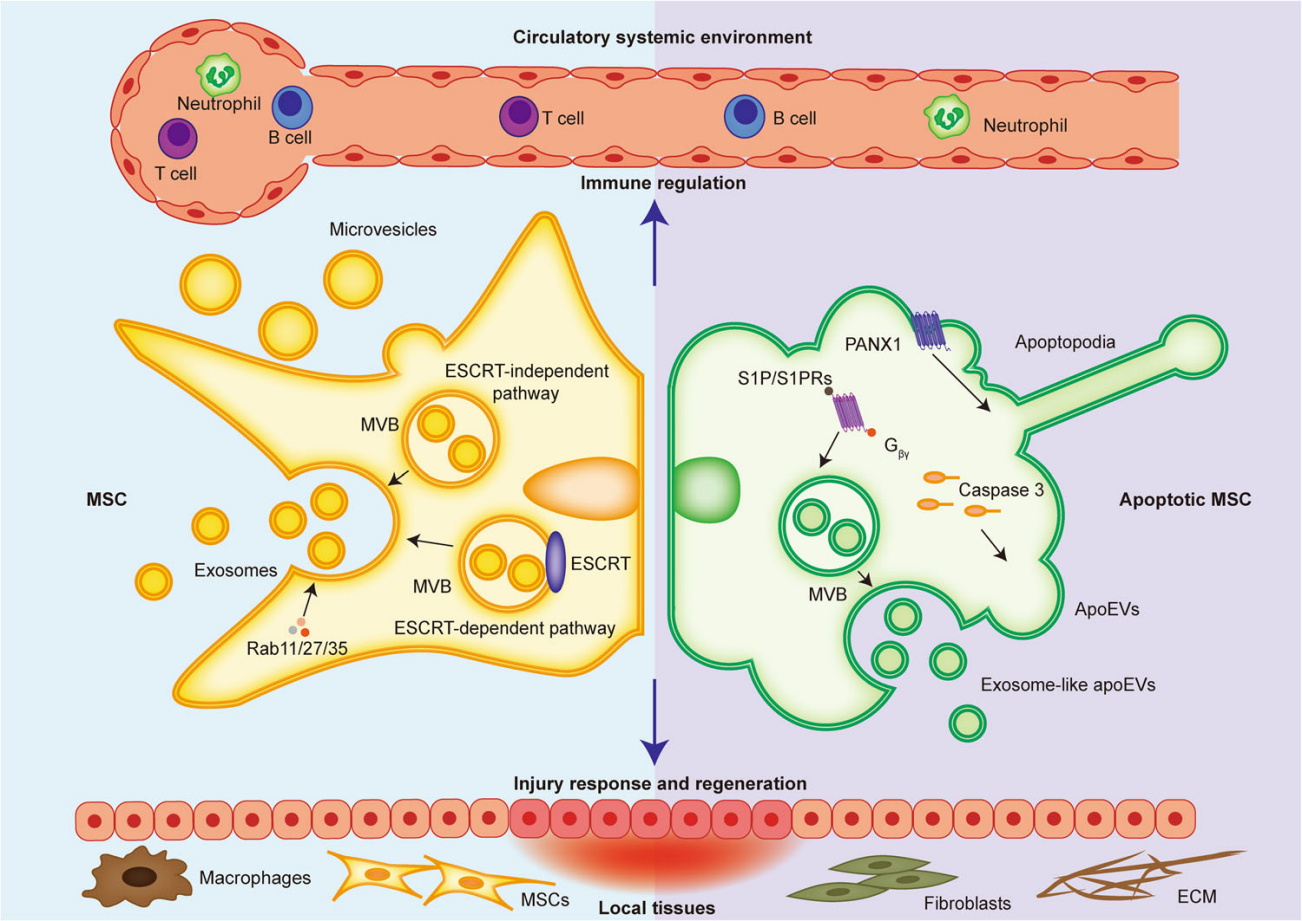
Generation and functionality of EVs from normal and apoptotic cells. From normal cells, exosomes are formed through exocytosis of endosomal membrane based on ESCRT-dependent and -independent mechanisms, which subsequently secrete exosomes *via* Rab11/27/35. Microvesicles are shed from the plasma membrane. In contrast, apoEVs are released from apoptotic cells based on multiple mechanisms including (S1P)/S1PRs- and Caspase 3-depended apoEV release as well as PANX1-controlled apoptopodia formation. On the one hand, EVs and apoEVs modulate immune responses in circulatory system. On the other hand, they are attributed to injury response and regeneration of local tissues.

ApoEVs secreted from apoptotic cells contain diverse bioactive factors which endow them with a key role in tissue homeostatic maintenance. Traditionally, the only known apoEV population was that of apoBDs (diameters range of 1000–5000 nm), although it is now understood that smaller apoEVs are simultaneously released from apoptotic cells^21^. As far as currently known, the production and secretion mechanisms of apoEVs share similarities with EVs but are also specifically characterized by apoptosis. As for EV formation, exosomes are generated in intracellular multivesicular bodies (MVBs) containing the several future exosomes called intraluminal vesicles (ILVs)^110^. MVBs primarily form through the invagination of plasma membrane and endosomal membrane based on ESCRT-dependent and -independent mechanisms, which subsequently secrete exosomes *via* Rab11/27/35-mediated exocytosis^110^ (Fig. 2). ApoEVs also contain exosomes-like subpopulation that is first formed in MVBs, but a featured molecular pathway is involved in apoEV release^111^. Specifically, cellular sphingosine1-phosphate (S1P)/S1PRs couple with G_βγ_ to stimulate the actin cytoskeleton during apoptosis execution, which orchestrate the progression of apoEV release^111^ (Fig. 2). Therefore, apoEV production are largely controlled by the apoptotic process, as further confirmed by Caspase 3 been shown as an upstream molecule for apoEV formation^112^. As revealed, blockade of caspase-activated pannexin 1 channels (PANX1) promotes generation of “beads-on-a-string” protrusion in monocytes, the produced apoEVs of which process are termed apoptopodia^112,113^. Apoptopodia controlled by the characterized mechanism represents a unique and novel way of apoptotic cell disassembly^112^ (Fig. 2). Notably, specific progression of apoptosis based on different cell types as well as physiological and pathological contexts contributes to different subtypes of apoEVs that contain distinct soluble metabolites, which also gives them a variety of functional properties^114^. Shotgun proteomics showed that apoEVs from human biliary epithelial cells of healthy control and cirrhosis patients possess significantly different proteomes^115^. It has been further validated that apoEVs contain a more active 20S proteasome core than that of apoBDs; this controls their immunogenic activity^116^. Whether the different production mechanisms of apoEVs contribute to potential functional discrepancies among apoEV subpopulations remains to be investigated.

Compared to the characteristics of exosomes or microvesicles, apoEVs have unique membrane molecular components, such as the apoptotic marker phosphatidylserine (PtdSer) and C1q, which also possess characterized miRnomes and proteomes based on specific content distribution during apoptosis^33,34,115,116^. The characteristics can be used as the standard for the identification, isolation, and purification of apoEVs. It has been reported that a set of convenient purification and identification procedures, such as gradient centrifugation, shotgun proteomics, and flow cytometry analysis, have been applied in experiments^33,115^. Considering the high output and large size of apoEVs, it is not necessary to go through tedious ultracentrifugation isolation steps, which is more convenient and rapid for apoEV-based cell-free therapeutics application^26,30,33^.

Despite of the heterogeneity, apoEVs are emergingly considered as physiological regulators, which not only help the apoptotic cell clearance but also contribute to immunomodulation and regeneration^21^. The endothelial cell-derived apoEVs which contain miR-126 induce recipient vascular cells to express and secrete the CXC chemokine CXCL12, resulting in the recruitment of progenitor cells for protection of vessels from atherosclerosis^25^. Moreover, a class of enriched interleukin 1 receptor antagonist (IL-1RA)-EVs secreted from MSCs are controlled by Fas, the receptor that initiates the extrinsic apoptotic pathway upon binding with FasL, and can accelerate wound healing^24^. Intriguingly, further evidence in a parabiosis mouse model, which connected green fluorescent protein (GFP) mice with apoptosis-deficient *Fas* mutant or *Caspase 3*^−*/*−^ mice, revealed that apoEVs participate in circulation to regulate distant MSCs^33^. It has also been reported that apoEVs induce CD4^+^ Treg responses and suppress CD8^+^ cytotoxic T-cell responses to exert antitumor immunity^117^. In addition, apoEVs from donor plasma with acute human immunodeficiency virus (HIV-1) infection specifically inhibit dendritic cells (DCs) *via* targeting CD44^118^. Taken together, these findings suggest that apoEVs are involved in multiple physiological contexts and pathological progressions, which may further contribute to regenerative and immunoregulatory therapeutic applications.

## Apoptotic cell and apoEV contributions to the therapeutic effects of MSCs

**Fig. 3:**
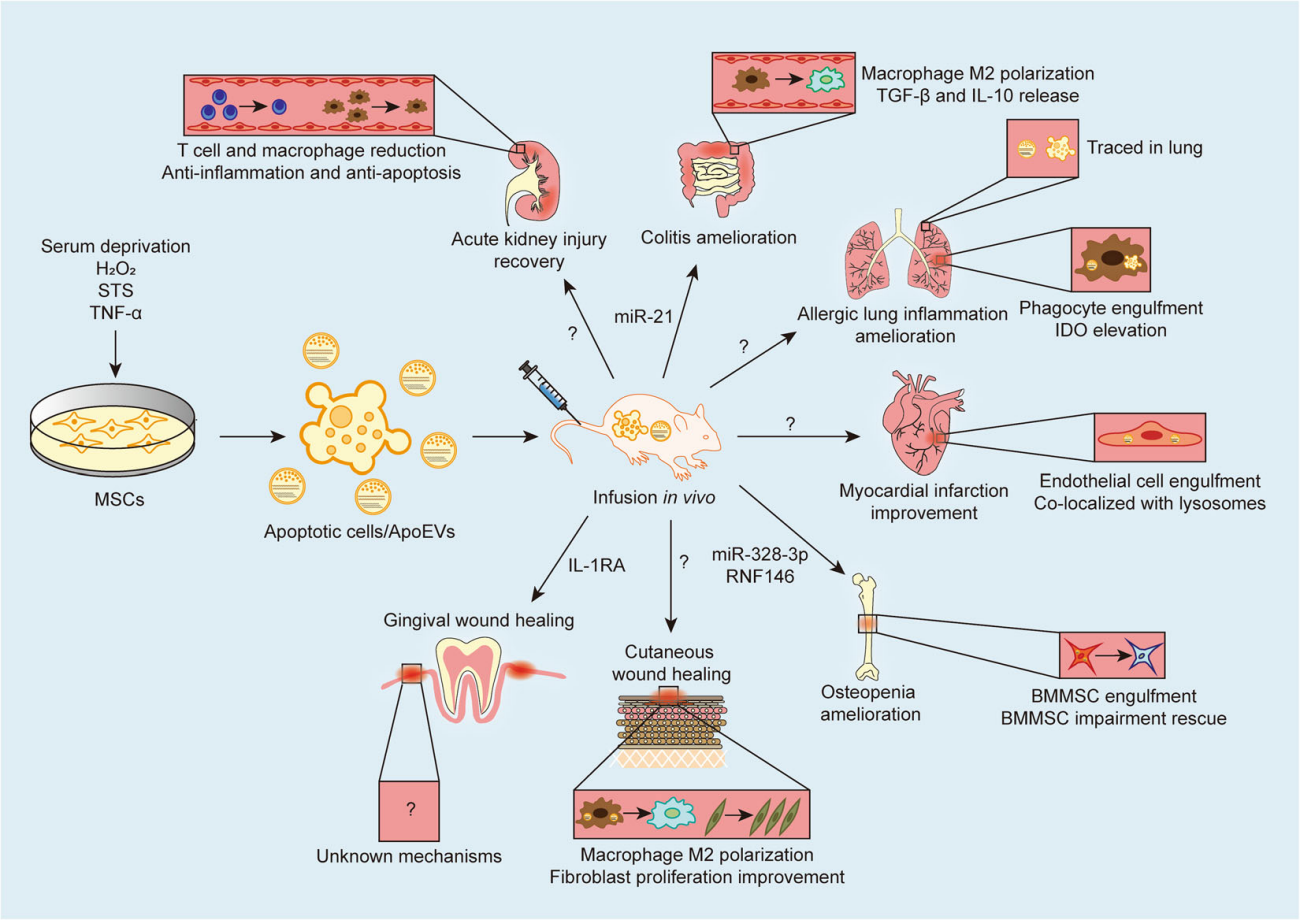
Apoptosis and apoEV contribution to the therapeutic effects of MSCs. Under the multiple exogenous apoptotic stimuli, MSCs in culture can be induced to apoptosis to form apoptotic cells and apoEVs, which are collected and infused to various disease models. ApoEVs transplanted or released by infused apoptotic cells have been demonstrated to carry bioactive proteins and miRNAs to recipient cells for tissue homeostasis maintenance and immunoregulation. For examples, infused apoptotic cells and apoEVs are traced in lung and engulfed by phagocytes, which possess potent capacities of immunomodulation in inflammatory insults. ApoEVs regulate the number of immune cells and promote macrophage M2 polarization in multiple diseases including acute kidney injury, the colitis, allergic lung inflammation, and cutaneous wound healing. Apoptotic cells and apoEVs can also be engulfed by endogenous MSCs and endothelial cells, contributing to the rescue of impaired stem cells and tissue regeneration. While the therapeutic effects of apoptotic cells and apoEVs are remarkable, the mechanisms underlying molecular delivery and potential interplays between donors and recipients remain elusive.

Since it was first noted that infused MSCs undergoing extensive apoptosis, apoptotic MSCs have been revealed as effective candidates for promoting immunoregulation and tissue regeneration in various diseases^28,29,33^ (Table 1 and Fig. 3). Adiministration of apoptotic adipose-derived MSCs (ADMSCs) has been demonstrated to significantly improve the survival rate of rats with sepsis syndrome relative to administration of healthy ADMSCs, further attenuating damage to multiple organs and reducing circulating TNF-α levels as well as those of oxidative and apoptotic biomarkers^31^ (Table 1 and Fig. 3). A further study has reported that apoptotic ADMSC infusion aids in the recovery from acute kidney injury (AKI), and tracing of apoptotic ADMSCs revealed engraftment in renal parenchyma^32^ (Table 1 and Fig. 3). Concerning their immunomodulatory capacity, transplanted apoptotic DPSCs significantly inhibit allergic lung airway inflammation in mouse GvHD^23^ (Table 1 and Fig. 3). Moreover, apoptotic human BMMSCs have also been traced to the lungs of GvHD mice, where they are engulfed by phagocytes to induce IDO production, resulting in the reduction of GvHD effector cell infiltration^29^ (Table 1 and Fig. 3).

**Table 1:** Application of apoptotic products in treating various disease models.

Term	Origin	Induction method	Quantity	Administration time	Route	Delivered molecule(s)	Animal model	Recipient species	Ref.
Apoptotic MSCs	Human ADMSCs	Serum deprivation	1.2 × 10^6^ cell-derived	30 min, 6 h, and 18 h after model establishment	*i.v*.	None	Sepsis syndrome	Rat	^31^
Apoptotic MSCs	Human ADMSCs	Serum deprivation	1.2 × 10^6^ cell-derived	30 min, 6 h, and 18 h after model establishment	*i.v*.	None	AKI	Rat	^32^
Apoptotic MSCs	Human DPSCs	H_2_O_2_	4.0 × 10^6^ cell-derived	Immediate after model establishment	*i.v*.	None	GvHD	Mice	^23^
Apoptotic MSCs	Human BMMSCs	Anti-Fas and granzyme B	1.0–2.5 × 10^6^ cell-derived	1 h after model establishment	*i.p. i.v*.	None	GvHD	Mice	^29^
MSC-derived EVs	Mice Gingival and skin MSCs	TNF-α	40 μg	1 d after model establishment	Submucosal injection	IL-1RA	Gingival wound	Mice	^24^
Apoptotic bodies	Mice BMMSCs	STS	50 μg	0 d, 3 d, and 7 d after model establishment	Local administration	None	Skin wound	Mice	^26^
Apoptotic bodies	Mice BMMSCs	STS	4.0 × 10^6^ ApoBDs	Once a week for 4 weeks	*i.v*.	miR-328-3p RNF146	Osteoporosis	Mice	^33^
Apoptotic bodies	Rat and mice BMMSCs	STS	100 μg	2 weeks after model establishment	Intramyocardial injection	None	MI	Rat	^30^
Apoptotic EVs	Mice thymocyte and Jurkat cells	UV-irradiated	20.0 × 10^6^ or 40.0 × 10^6^ cell-derived	1 d before model establishment	*i.p*.	None	Colitis	Mice	^22^
Chimeric apoptotic bodies	Mice T-cell membrane with mesoporous silica nanoparticles	STS	100 μg	3 d, 5 d, 7 d, and 9 d after model establishment	*i.v*.	miR-21 and curcumin	Colitis and cutaneous inflammation	Mice	^34^

*AKI* acute kidney injury, *ADMSCs* adipose-derived mesenchymal stem cells, *ApoBDs* apoptotic bodies, *BMMSCs* bone marrow mesenchymal stem cells, *DPSCs* dental pulp stem cells, *EVs* extracellular vesicles, *GvHD* graft-versus-host disease, *IL-1RA* interleukin 1 receptor antagonist, *i.p*. intraperitoneal injection, *i.v*. intravenous injection, *MI* myocardial infarction, *miR* microRNA, *MSCs* mesenchymal stem cells, *STS* staurosporine, *TNF-α* tumor necrosis factor-α.

The potiential capacity of MSC-apoEVs to mediate tissue regeneration and immunomodulation in vivo has also been proposed. Under the apoptotic stimulus of TNF-α, transplanted MSC-derived EVs have been demonstrated to promote gingival wound healing^24^ (Table 1 and Fig. 3). A recent study has also validated that MSC-derived apoEV infusion promotes cutaneous wound healing through polarizing surrounding macrophages to facilitate migration and proliferation of fibroblasts^26^ (Table 1 and Fig. 3). It is notable that delivery of apoEVs produced by allogeneic MSCs remarkably rescues the osteopenic phenotype in the apoptosis-deficient *Fas* mutant and *Caspase 3*^*−/−*^ mouse models^33^. Infusion of exogenous MSC-apoEVs is also effective in ameliorating osteoporosis in estrogen-deficient ovariectomized (OVX) mice^33^. Mechanistic investigations showed that infused apoEVs are engulfed by recipient-impaired MSCs in vivo, with effects mediated by concerted transfer of miR-328-3p and ubiquitin ligase RNF146 to activate canonical WNT signaling for endogenous MSC recovery^33^ (Table 1 and Fig. 3). In heart injury, transplanted MSC-apoEVs regulate autophagy in cardiac endothelial cells, which enhance angiogenesis and improve cardiac functional recovery in a myocardial infarction model^30^. Mechanistically, infused MSC-apoEVs have been revealed to facilitate the translocation of transcription factor EB (TFEB) from lysosomes to the nucleus, which regulates the target genes associated with autophagy and lysosomal biogenesis^30^ (Table 1 and Fig. 3).

As for their immunoregulatory properties, apoEVs can modulate T-cell responses and macrophage signaling cascades in vivo, ameliorating experimental colitis^22^. Mechanistically, PtdSer located on the surface of apoEVs stimulates macrophages to upregulate transforming growth factor-β (TGF-β) production and reduces their forkhead box O3 (FOXO3) level^22^ (Table 1 and Fig. 3). A recent study has also used chimeric apoEVs, which are established by loading apoEV membranes with nanoparticles and anti-inflammatory agents, to actively target macrophages to promote M2 polarization^34^. Animal experiments confirmed the chimeric apoEV have remarkable therapeutic effects in treating cutaneous inflammation and colitis^34^. These findings collectively suggest that apoEVs are key mediators of MSCT and that apoEV administration is a promising cell-free therapeutic strategy.

As reported, the preparation processes of apoptotic MSCs did not go through isolation and purification, the therapeutic effects of apoptotic MSC transplantation may be attributed to the existence of apoEVs. The therapeutic effects of apoEVs, as well as apoptotic cells, are mainly relied on the phagocytosis of recipient cells. Although there are few reports about the characteristic comparison between apoptotic MSCs and apoEVs, it has been demonstrated that apoEVs contain a more active 20S proteasome core than that of apoBDs^116^. Therefore, the transfer of biological signals in the form of apoEVs may be a unique and specific way.

## Conclusions and perspectives

MSCT has achieved great advances in treating various diseases and realizing tissue regeneration and immunomodulation^8,9^, although the challenge of how to precisely control therapeutic effects of MSCs remains to be addressed. As recent studies have reshaped our perception about apoptosis and revealed it to be critically involved in multiple physiological and pathological contexts, infusion of MSC-derived apoEVs has demonstrated remarkable therapeutic effects and emerged as a novel and potential cell-free therapeutic^22,26,30,33^. Considering the significant therapeutic effects of apoEV transplantation and the phenomenon of autonomously tissue regeneration caused by endogenous apoptotic stimuli, it is proposed that MSCs could contribute to systemic and local tissue homeostasis maintenance through autonomous apoEV production^100–106^. Intriguingly, accumulating studies have recently revealed that autophagic inducement enhances MSC properties in vitro, particularly in differentiation potential and its immunoregulation capacity^119–121^. It has also been demonstrated that the MSC-derived inflammasomes managed by pyroptosis promote inflammatory response in vivo^122^. As known, immunogenic intracellular materials are released from cells under necroptosis execution, which activate immune systems and trigger extensive inflammatory response in organisms^123^. Accordingly, transplanted MSCs might undergo death under diverse physiological and pathological contexts, such as autophagic cell death, the necroptosis or the pyroptosis for the contribution of tissue homeostasis maintenance. As the results of parabiosis mouse model shown, apoEVs participate in the circulation^33^. Questions of whether EVs produced by multiple cell death processes have differential regulatory and therapeutic effects in circulation are also interesting but still unsolved in this field.

Many studies have shown the chemotaxis of infused MSCs toward injured or inflammation sites, the issue of whether apoEVs possess specific tissue chemotaxis remains elusive. As known, infused MSCs exert immense therapeutic effects through direct effects of engraftment and differentiation, as well as indirect effects of paracrine mechanism and apoptosis execution, while transplanted apoEVs carry characterized miRnomes and proteomes to exert therapeutic effects^115,116^. It is notable that the generation of apoEVs, particularly regarding whether apoptosis execution of transplanted MSCs occurs before or after engraftment and migration, remains elusive. Albeit not fully understood, the targeting of apoEVs is closely related to unique membrane components, such as PtdSer and C1q^33,34^, which is an important matter and worthy of investigation in this field. Extensive experiments should be performed to investigate specific targeting of infused apoEVs and the underlying mechanisms.

Compared to MSCT, apoEV therapy possesses advantages including low immunogenicity, easy storage of reagents, reduced coagulation risk, and amenability to engineering for drug delivery. Key theranostic issues, such as heterogeneity, storage condition, quality control as well as standardization of apoEVs in induction and purification, still remain to be investigated. The realization of this paradigm shift from living MSCs to apoptotic materials will surely provide innovative and promising guidance for de novo organ regeneration and immunoregulation in future translational medicine.
